# Black ethnicity predicts better survival on dialysis despite greater deprivation and co-morbidity: a UK study 

**DOI:** 10.5414/CN108247

**Published:** 2014-07-02

**Authors:** Nicholas Cole, Michael Bedford, Andrew Cai, Chris Jones, Hugh Cairns, Satish Jayawardene

**Affiliations:** King’s College Hospital Renal Unit, Denmark Hill, London, UK

**Keywords:** end-stage renal disease, dialysis, ethnicity, mortality, survival, diabetes, deprivation

## Abstract

Background: Observational studies from the United States have identified black race as conferring a survival advantage on dialysis. This study represents the first large single-center study from a UK dialysis unit examining the outcome of ethnic minorities on renal replacement therapy (RRT). Methods: A retrospective analysis of all patients of white or black race initiating RRT at King’s College Hospital Renal Unit, London, between 1996 and 2008 was performed. A total of 1,340 patients were studied, of which 952 (71%) were of white race, and 388 (29%) were of black race. Kaplan-Meier survival curves, the log rank test and Cox’s proportional hazard models were used to compare survival between groups. Results: The results revealed black ethnicity to be associated with a significant survival benefit on dialysis. This was the case even after adjustment for age, gender, diabetes, transplantation, and deprivation. In those patients not transplanted, black race conferred a hazard ratio (HR) of 0.51 (95% CI 0.41 – 0.63) over 5 years. Conclusions: This study provides evidence for a lower mortality rate amongst black patients on dialysis in comparison with their white counterparts in the UK. The reasons behind this remain poorly understood but a lower incidence of cardiovascular disease in black patients and more kidney-limited disease may be important.

## Introduction 

Patient survival on dialysis is substantially reduced compared with the general population and understanding the factors contributing to this mortality is of importance if outcomes are to be improved. Observational studies from the United States (US), as well as Canada and The Netherlands, have identified black race as conferring a survival advantage on dialysis [1, 2, 3, 4, 5]. After adjustment for age, gender, and primary renal diagnosis, the US Renal Data System (USRDS) reports a 5-year survival probability of 0.38 for black patients on dialysis in comparison to 0.32 for white patients [1]. This survival advantage is seen for patients both on peritoneal dialysis and hemodialysis, and disappears following transplantation (where survival rates are comparable and may even be lower for black patients) [6]. The lower mortality rates seen on dialysis are curious, given that black ethnicity is associated with a number of predictors of adverse health outcomes and is associated with higher mortality rates in the general population and amongst those with early chronic kidney disease (CKD) [7]. 

We set out to investigate whether or not this survival paradox was present in our dialysis population. The United Kingdom (UK) differs from the US in terms of both its population and its healthcare delivery, and it is not certain that the studies discussed above are applicable to the UK dialysis population. There is recent data from the UK demonstrating a lower mortality in black patients compared to white patients with CKD in a primary care setting [8]. However, two studies from UK renal units have failed to show a significant survival advantage for black patients on dialysis. These studies were from predominantly white and Indo-Asian communities with comparatively small black populations [9, 10]. More recently, Roderick et al. [11] examined UK Renal Registry data and found significantly better survival in black and South Asian patients on dialysis compared to Caucasians, even after adjustment for age, sex, renal diagnosis, and social deprivation. However, such a study could be confounded by regional variations in health status and dialysis provision across a country in which the black community remains very localized and makes up only a small proportion of the dialysis population. Bearing this in mind, the King’s College Hospital (KCH) Renal Unit in London is well placed to further investigate because it has the highest prevalence of black patients on RRT in the UK (32% of the dialysis population) [12]. 

## Methods 

### Study design and data collection 

This study was a retrospective analysis of patients of white or black race starting RRT at KCH Renal Unit, London. The data was collected prospectively between 1996 and 2008. Patient demographics, mode of RRT, and primary diagnosis were later extracted from the renal unit database (Renalware). Ethnicity data was available for all patients having been entered at the time of patient presentation. Only patients of white or black ethnicity were included in the study, with Asian populations being excluded due to low numbers. 

The length of time patients had spent on modes of RRT, as well as co-morbidities, were manually collected from electronic patient notes. The cause of end-stage renal disease (ESRD) had been coded by a consultant physician. Cardiovascular disease (CVD) was defined as documented evidence of one or more of ischemic heart disease (myocardial infarction, angina or coronary artery stent or grafting), cerebrovascular disease (stoke or transient ischemic attack), or peripheral vascular disease including artery stenting or bypass. Diabetes was analyzed as a cause of ESRD because this group represented the majority of those with established diabetes. Mortality between both groups was compared according to the most recent modality of RRT. 

### Social deprivation index 

Deprivation scores were calculated matching patient postcodes to area scores of the UK Index of Multiple Deprivation (IMD) 2007 [13]. The IMD 2007 brings together 7 domains and 37 different indicators that cover specific aspects or dimensions of deprivation. The domains are: income, employment, health and disability, education, skills and training, barriers to housing and services, living environment, and crime. These are weighted and combined to create the overall IMD 2007 score. A higher score indicated that an area was more deprived. 

**Statistical analysis**

Analysis of variables included χ^2^- and t-test (Mann-Whitney for non-parametric data) for categorical and continuous variables. Analysis of variance (ANOVA) was used to compare longitudinal trends within individuals. Kaplan-Meier survival curves, the logrank test, and Cox’s proportional hazard models (hazard ratio, HR) were used to compare survival between groups, with the impact of patient factors on survival assessed using univariate and multivariate analysis. 

## Results 

### Demographics 

1,340 patients of either white or black race were identified to have started RRT at King’s College Hospital Renal Unit in the 12 years between 1996 and 2008. Of these, 952 (71%) were of white ethnicity, and 388 (29%) were of black ethnicity. The study population characteristics, separated by ethnicity, are summarized in Table 1. 

Black patients were significantly younger than their white counterparts at time of initiating RRT, by an average of 8 years. They also had significantly higher social deprivation scores, reflecting greater deprivation. Significantly more ESRD in black patients was attributed to both diabetes and hypertension compared to white patients. Conversely, there was a lower prevalence of renovascular disease and CVD. The proportion of black and white patients transplanted was similar, although black patients were over-represented on the transplant list in comparison to white patients (51% vs. 29%, respectively). 

### Survival according to ethnicity 

**Figure 1.**
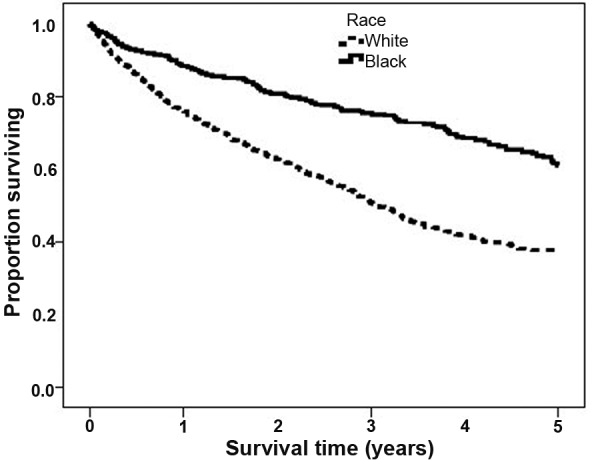
Five-year survival in dialysis patients by ethnicity (adjusted for age, gender, diabetes, transplantation, and deprivation).

Even after adjustment (for age, gender, diabetes, transplantation, and deprivation), black ethnicity was associated with a significant survival benefit on dialysis over 5 years (Figure 1) (Table 2). In those patients not transplanted, black race conferred a HR of 0.51 (95% CI 0.41 – 0.63). Over a longer period of 10 years, survival for dialysis patients not transplanted was 9% for white patients and 26% for black patients (p < 0.001, data not shown). In transplanted patients, there was no difference in survival between both ethnic groups. 

### Effect of diabetes and deprivation 

**Figure 2.**
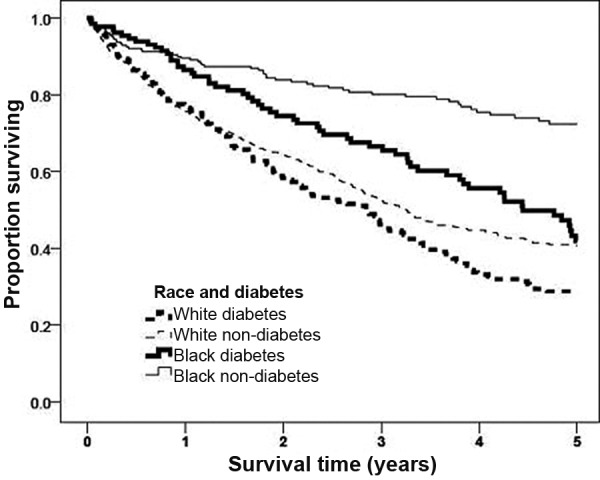
Five-year survival by ethnicity and diagnosis of diabetes (adjusted for gender, diabetes, transplantation, and deprivation).

Black patients on dialysis had a lower mortality rate despite greater social deprivation and a higher prevalence of diabetes and ESRD attributed to diabetes. Deprivation was not a significant determinant of mortality (Table 2). As expected, diabetic ESRD was associated with a poorer outcome in both ethnic groups compared to non-diabetic ESRD, but there was a suggestion that this effect was more pronounced in the white population (Figure 2). For those of white ethnicity, non-diabetic ESRD was associated with a HR of 0.79 (p = 0.06, 95% CI 0.62 – 1.01), whereas for those of black ethnicity, the HR was 0.88 (p = 0.58; 95% CI 0.57 – 1.37). 

## Discussion 

This is the first single-center study from the UK to have demonstrated a significantly lower mortality in black dialysis patients in comparison to white dialysis patients. The survival advantage seen in black patients on dialysis outweighed the potential adverse effects of greater social deprivation and a higher prevalence of diabetes and hypertension found within this population. These findings will be discussed below, together with the potential insights gained. 

### Diabetes 

The higher prevalence of diabetic ESRD within the black population would be expected to have a negative effect on overall mortality, given that diabetes is a known risk factor for CVD and sepsis. However, the survival rate of black dialysis patients with diabetic ESRD was at least equal to white patients with non-diabetic ESRD. Furthermore, there was a trend towards diabetic ESRD being associated with greater mortality in white patients compared to black patients. This supports the view that there is an unknown factor that “protects” black patients on dialysis. 

### Social deprivation 

In the general population, social deprivation is widely acknowledged as being associated with adverse health outcomes. This is believed to reflect disparities in health behaviors, education and access to healthcare services. However, in this study the greater deprivation amongst black dialysis patients compared to their white counterparts did not translate to a poorer outcome. This finding has also been reported in other studies and suggests that there are more significant determinants of survival [11, 14]. 

### Transplantation 

The proportion of black and white patients transplanted was similar in the two groups and did not account for the survival difference on dialysis. The proportion of the dialysis population on the transplant waiting list who were transplanted was 62% for white patients and 37% for black patients. This is consistent with USRDS and National Health Service Transplant data showing reduced access to transplantation amongst the black population [1, 15]. The reasons behind this over-representation on the list are likely multi-factorial, and include differences in blood and tissue-types, as well as lower donation rates amongst the black population. However, it is also interesting to consider whether or not it also reflects a more healthy population. Given the survival advantage demonstrated in this study, it is plausible that a greater proportion of black patients are medically fit for listing. There is limited evidence for this either way. USRDS data for first-time listed patients at 3 years of listing, showed that 11.7% of white patients had died waiting compared to 11.1% of black patients [1]. More data may lead to further insights. 

### Co-morbidity 

CVD is the most significant cause of mortality amongst the dialysis population, accounting for up to 40% of deaths [1]. In keeping with a number of other studies, we found significantly lower rates of CVD (as well as renovascular disease) in the black ESRD population compared with the corresponding white population [16, 17, 18]. Black ethnicity has been shown to be associated with a lower prevalence of atherosclerotic CVD at the start of dialysis and a lower incidence of new or recurrent cardiovascular events after starting dialysis [17]. The survival advantage for black patients on dialysis may be due, at least in part, to a lower risk of CVD. 

This is surprising given the higher incidence of diabetes and hypertension within the same population, both of which are known to be cardiovascular risk factors both in the general population and amongst those on dialysis [19]. Once again, there may be environmental and/or genetic factors that “protect” the black population from developing CVD. These have yet to be identified although higher high-density lipoprotein levels may contribute [20]. 

### Primary renal diagnosis 

Differences in primary renal diagnoses between both ethnic groups have been demonstrated in this study. In the white dialysis population, the significantly higher rates of renovascular disease, amyloid, and myeloma may account for the higher mortality, via poor prognosis of the underlying disorder or its associated conditions. For example, amyloidosis and myeloma caused 6.5% of ESRD in the white population and 2% in the black population. This could account for up to 20% of the difference in mortality observed. On the other hand, a higher proportion of black patients had ESRD that was attributed to hypertension. Hypertension-attributed renal failure in this group has been strongly linked with the *ApoL1* gene [21, 22, 23]. It could be that *ApoL1*-associated nephropathies in this group are more likely to cause renal-limited disease with fewer cardiovascular complications than in white patients with hypertension. 

### Study limitations 

There are limitations to this study. It is limited by the fact that it represents data from a single renal unit population with relatively small numbers of patients. There are also likely to be confounding factors present that were not investigated. For example, we did not further explore differences in RRT delivery or collect data on biochemical markers such as hemoglobin and albumin. Furthermore, not all causes of ESRD were biopsy-confirmed and so diagnostic accuracy cannot be confirmed. Finally, we also used geo-code data to assess social deprivation and this is known to have limitations in that it may not accurately represent the lives of individuals. 

### Summary 

This study provides evidence for a lower mortality rate amongst black patients on dialysis in comparison with their white counterparts in the UK. The reasons behind this survival paradox are poorly understood but are likely to be multi-factorial. There may be genetic or environmental factors that confer a survival advantage, in which case their identification could lead to improved outcomes for all patients with ESRD. Alternatively, further understanding may highlight inequalities in healthcare delivery that need to be addressed. 

## Conflict of interest 

None to declare. 

**Table 1.** The clinical and demographic characteristics of incident ESRD patients.^a^

	White (952 (71%))	Black (388 (29%))	Total (1,340 (100%))	p-value
Male gender	583 (61%)	229 (59%)	812 (61%)	0.45
Mean age in years (SD)	62 (16)	54 (16)	60 (16)	< 0.001
Co-morbidity:				
Diabetes	314 (33%)	169 (44%)	483 (36%)	< 0.001
Cardiovascular disease	495 (52%)	171 (44%)	657 (49%)	0.027
Cause of ESRD:				
Diabetes	248 (26.1%)	132 (34.0%)	380 (28.4%)	0.003
Diagnosis unconfirmed	172 (18.1%)	81 (20.9%)	253 (18.9%)	0.36
Glomerulonephritis	169 (12.8%)	46 (11.9%)	168 (12.5%)	0.09
Hypertension	69 (7.3%)	78 (20.1%)	147 (11.0%)	< 0.001
Pyelonephritis	107 (11.3%)	24 (6.2%)	131 (9.8%)	0.06
Renovascular disease	83 (8.7%)	5 (1.3%)	88 (6.6%)	< 0.001
Polycystic kidneys	54 (5.7%)	9 (2.3%)	63 (4.7%)	0.23
Myeloma	41 (4.3%)	6 (1.5%)	47 (3.5%)	0.07
Other	34 (3.6%)	5 (1.3%)	39 (2.0%)	0.42
Amyloid	21 (2.2%)	2 (0.5%)	23 (1.7%)	0.09
Mean years on RRT by modality (SD):				
Hemodialysis	1.9 (2.3)	2.5 (2.1)	2.1 (2.2)	0.001
Peritoneal dialysis	1.7 (1.5)	1.7 (1.7)	1.7 (1.6)	0.66
Transplant	4.6 (3.5)	4.4 (3.6)	4.7 (3.5)	0.67
UK IMD score (SD)	24 (14)	35 (11)	28 (14)	<0.001
Transplant listed	276 (29%)	198 (51%)	474 (35%)	<0.001
Transplanted	170 (18%)	74 (19%)	244 (18%)	0.61

ESRD = end-stage renal disease; RRT = renal replacement therapy; SD = standard deviation; UK IMD = United Kingdom Index of Multiple Deprivation. ^a^Data are shown as number (percentage) unless otherwise specified.

**Table 2.** Factors predicting mortality at 5 years after starting RRT using multivariate Cox’s proportional hazard model.

	Hazard ratio	95% CI		p-value
		Lower	Upper	
Deprivation score	1.00	0.99	1.00	0.78
Age at RRT start (per year older)	1.02	1.02	1.03	< 0.001
Black ethnicity	0.51	0.40	0.64	< 0.001
Diabetic cause of ESRD	1.63	1.07	2.50	0.02
Female gender	1.06	0.88	1.28	0.48
Never transplanted	7.17	4.29	11.9	< 0.001

ESRD = end-stage renal disease; RRT = renal replacement therapy.
